# Robust disruptions in electroencephalogram cortical oscillations and large-scale functional networks in autism

**DOI:** 10.1186/s12883-015-0355-8

**Published:** 2015-06-27

**Authors:** Sean Matlis, Katica Boric, Catherine J. Chu, Mark A. Kramer

**Affiliations:** Graduate Program in Neuroscience, Boston University, 677 Beacon st., Boston, MA 02215 USA; Department of Neurology, Massachusetts General Hospital, 175 Cambridge St., Ste 340, Boston, MA 02114 USA; Harvard Medical School, Boston, MA 02115 USA; Department of Mathematics and Statistics, Boston University, 111 Cummington Mall, Boston, MA 02215 USA

**Keywords:** ASD, EEG, Functional networks, Biomarker, Classification, Autism, Power spectra, Validation

## Abstract

**Background:**

Autism spectrum disorders (ASD) are increasingly prevalent and have a significant impact on the lives of patients and their families. Currently, the diagnosis is determined by clinical judgment and no definitive physiological biomarker for ASD exists. Quantitative biomarkers obtainable from clinical neuroimaging data – such as the scalp electroencephalogram (EEG) - would provide an important aid to clinicians in the diagnosis of ASD. The interpretation of prior studies in this area has been limited by mixed results and the lack of validation procedures. Here we use retrospective clinical data from a well-characterized population of children with ASD to evaluate the rhythms and coupling patterns present in the EEG to develop and validate an electrophysiological biomarker of ASD.

**Methods:**

EEG data were acquired from a population of ASD (*n* = 27) and control (*n* = 55) children 4–8 years old. Data were divided into training (*n* = 13 ASD, *n* = 24 control) and validation (*n* = 14 ASD, *n* = 31 control) groups. Evaluation of spectral and functional network properties in the first group of patients motivated three biomarkers that were computed in the second group of age-matched patients for validation.

**Results:**

Three biomarkers of ASD were identified in the first patient group: (1) reduced posterior/anterior power ratio in the alpha frequency range (8–14 Hz), which we label the “peak alpha ratio”, (2) reduced global density in functional networks, and (3) a reduction in the mean connectivity strength of a subset of functional network edges. Of these three biomarkers, the first and third were validated in a second group of patients. Using the two validated biomarkers, we were able to classify ASD subjects with 83 % sensitivity and 68 % specificity in a post-hoc analysis.

**Conclusions:**

This study demonstrates that clinical EEG can provide quantitative biomarkers to assist diagnosis of autism. These results corroborate the general finding that ASD subjects have decreased alpha power gradients and network connectivities compared to control subjects. In addition, this study demonstrates the necessity of using statistical techniques to validate EEG biomarkers identified using exploratory methods.

**Electronic supplementary material:**

The online version of this article (doi:10.1186/s12883-015-0355-8) contains supplementary material, which is available to authorized users.

## Background

Autism Spectrum Disorders (ASDs) are a group of disorders characterized by impairment in communication, and social interaction, rigidity of interests, and repetitive stereotypical behaviors [1]. First characterized as a behavioral disorder in 1943, the diagnosis of autism appears to be increasing, from less than 3 per 10,000 in the 1970’s to more than 30 per 10,000 in the 1990s [2]. In 2012, the Centers for Disease Control reported a frequency of 1 in 88 children [3, 4]. The symptoms and severity of ASD varies significantly, and mild symptoms or those masked by other handicaps may go unrecognized. Appropriate treatments, especially in children, have been found to lead to improved prognosis [5, 6], which has motivated the search for biomarkers that can assist identification of ASD in children [7–9].

Because ASDs are defined by behavioral traits, the diagnosis relies on questionnaires and observation. Multiple genetic and biological risk factors have been identified [6], however isolating a common metabolic or genetic pathophysiology leading to the ASD phenotype has proven difficult [10]. An alternate approach is to measure differences in observable brain function. There is a growing consensus that ASD is characterized by an impairment in the communication between brain areas, rather than a deficit in a localized brain region [11–21]. Advances in neuroimaging and signal processing allow for inference and analysis of functional brain networks, which represent the dynamic relationships between activity recorded in different brain regions [22]. Analytical methods derived from the field of graph theory allow measurement and comparison of functional networks in health and disease [13, 23–25]. While functional magnetic resonance imaging (fMRI) has received much attention as a tool for investigating functional brain networks derived from hemodynamic signals, the scalp electroencephalogram (EEG) provides several distinct advantages as it is less expensive, less intrusive, less affected by head motion artifacts, and provides a direct measure of brain voltage activity. In addition, the EEG provides exquisite temporal resolution, allowing for investigation of cortical rhythms, as characterized in the power spectra, which have also been associated with brain function and dysfunction [26]. Thus, EEG provides benefits as a complimentary tool to fMRI, with advantages for use in children with autism in particular.

Despite these powerful tools, both EEG and fMRI research of ASD have so far produced varied and sometimes contradictory results. Arguably the most consistent finding is a reduced level of coupling in activity of ASD subjects between ‘long range’ areas [12, 16, 19, 27–41], though even this finding is not universal [42–50]. ‘Short range’ coupling has been found to be both greater [12, 34, 37, 39] and lower [27–29, 31, 32, 44, 47, 48, 51, 52] in ASD subjects compared to control subjects. While a number of studies report seemingly similar results, all previously reported studies have used exploratory methods with results found using different techniques, limiting interpretation and cross-study validation. Post-analysis validation has not been employed in existing studies of ASD biomarkers, but is an important step to separate statistically robust findings from chance observations. Without a consensus on particular measures associated with ASD or its individual characteristics, the search for definitive, physiological biomarkers of ASD continues.

In this study, we use spectral and functional network analysis of clinical EEG data recorded from a population of children to propose a cortical biomarker for autism. We first use an exploratory dataset of age-matched (4–8 years) ASD and neurotypical children to develop hypotheses based on analysis of power spectral features and measures of functional network connectivity. From the exploratory group of subjects, we derive the following hypotheses: 1) The ratio of the power of the posterior alpha rhythm (8–14 Hz) peak to the anterior alpha rhythm peak is significantly lower in ASD than control subjects. 2) The functional network density is lower in ASD subjects than control subjects. 3) A select group of edges provide a more sensitive and specific biomarker of ASD. We then test these hypotheses in a validation dataset of subjects and show that both the first and third hypotheses, but not the second, are validated. These results provide a validated study for EEG biomarkers of ASD based on changes in brain rhythms and functional network characteristics.

## Methods

### Subjects and EEG recordings

All subjects ages 4–8 years diagnosed with ASD by a specialist in child neurology, child psychiatry, or developmental pediatrics, and with an EEG obtained between 2/1/2002-4/1/2011 in the neurophysiology unit at Massachusetts General Hospital were retrospectively identified. In order to reduce variability in the ASD group, subjects diagnosed with epilepsy or found to have epileptiform activity on EEG were excluded from analysis. For control data, subjects age 4–8 years with normal EEG recordings (as defined by clinical electroencephalographers independent from this study) were retrospectively identified from recordings performed at Massachusetts General Hospital between 2/1/2002 and 4/1/2011. Clinical chart review was performed and only those children with documented normal neurodevelopment and non-epileptic events without known EEG characteristics were included in the control group for analysis. For both the ASD group and control group, neurodevelopmental status was determined from chart review of the clinical assessments just prior to or following the EEG recording. Active medications at the time of EEG include in the ASD training data, one subject was taking 0.1 mg of Clonidine and one subject was taking 20 mg of Ritalin, and in the ASD validation data one subject was taking 0.05 mg Clonodine and 0.5 mg Risperdal. Of the control subjects, no medications were taken in the training group, and in the validation group one subject was taking 50 mg of Amitriptyline, and one subject took 0.05 mg of Clonidine prior to the EEG. Twenty-seven children with ASD (25 M and 2 F) and fifty-five controls (29 M and 26 F) were included for analysis. In the ASD training group (defined below), one subject had ADHD and one reported headaches. In the ASD validation group (defined below), three subjects had ADHD - one of which had depression, and one of which had tics – while one other subject had only tics, another subject had ADD, and another subject had anxiety. Of the 55 neurotypical controls, 13 had migraines or other headache syndromes, 9 had a syncopal event, 8 had tics, 4 had anxiety, 1 had sleep apnea, 1 had breath holding spells, and 1 had essential tremor. Although formal scales of ASD severity were not used in this population, chart review of physical exam and clinical assessments was performed retrospectively by a board certified child neurologist (CJC). Using the DSM V criteria, severity was estimated as follows: in the training group of thirteen ASD subjects: eight mild, four moderate, and one severe ASD. In the validation group of fourteen ASD subjects: five mild, three moderate, and six severe ASD.

In an effort to identify a clinically feasible and relevant EEG biomarker for ASD, we utilized routine EEG recordings following standard clinical recording techniques. All children were given the same instructions prior to the evaluation, including recommendations for mild sleep deprivation (awaking the child 2–4 h prior to regular morning arousal). In our dataset, sleep was recorded in 18/27 ASD subjects and 45/55 healthy controls. In all cases with a sleep recording, sleep onset was within 40 min of the start of the EEG recording session. In all cases, the wake EEG was obtained first and a posterior dominant rhythm was obtained during a period of quiet restfulness with eyes closed. For recordings of quiet wakefulness, patients were recorded in a quiet room without active stimulation.

Recordings included electrooculogram (two channels), scalp EEG (19 Ag/AgCl electrodes placed according to the 10–20 international system: FP2, F4, C4, P4, O2, F8, T4, T6, Fz, Cz, Pz, Fp1, F3, C3, P3, O1, F7, T3, and T5) and electrocardiogram using a standard clinical recording system (Xltek, a subsidiary of Natus Medical). Signals were sampled at 200, 256, 500 or 512 Hz and stored on a local server. Analysis of the data from these subjects was performed retrospectively under protocols approved and monitored by local Institutional Review Boards according to the National Institutes of Health guidelines.

Prior to analysis, subject datasets were divided into two groups, one group for exploratory analysis and hypothesis creation and a second group for hypothesis validation. The subjects in each group were selected to preserve approximately similar age distributions in each group (Table 1). In this way, hypotheses generated in the first group were tested, and validated or disputed in the second group, thereby controlling for spurious findings due to type I error. EEG recordings were manually reviewed by an experienced electroencephalographer (CJC) and large movements and muscle artifact removed. Wake and non-rapid eye movement (NREM) sleep intervals were identified by visual analysis as per standard criteria [53]. Only patients with at least 100 s of artifact-free EEG data were included in the exploratory group (13 ASD, 24 Control) and validation group (14 ASD, 31 Control).

**Table 1:** Patient demographics

Group	Age 4	Age 5	Age 6	Age 7	Age 8	Total
ASD Training	2 M	2 M, 1 F	4 M	3 M	1 M	12 M, 1 F
ASD Validation	1 M	2 M	4 M	5 M, 1 F	1 M	13 M, 1 F
Control Training	3 F	4 M, 1 F	5 M, 1 F	3 M, 3 F	1 M, 3 F	13 M, 11 F
Control Validation	4 M, 2 F	3 M, 3 F	4 M, 1 F	2 M, 5 F	3 M, 4 F	16 M, 15 F

**Table 2:** Edges chosen for “mask”

ASD mask network edges	Fp1-F3/Fp1-F7; F3-C3/F7-T3; C3-P3/C4-P4; C3-P3/T3-T5; P3-O1/P4-O2; P3-O1/T5-O1; F4-C4/F8-T4; F4-C4/T4-T6; C4-P4/P4-O2; C4-P4/T4-T6; C4-P4/T6-O2; C4-P4/Cz-Pz; P4-O2/T5-O1; P4-O2/T6-O2; T5-O1/T6-O2; T4-T6/Cz-Pz
Control mask network edges	Fp1-F3/Fp2-F4; Fp1-F3/Fp1-F7; Fp1-F3/F7-T3; Fp1-F3/Fp2-F8; Fp1-F3/F8-T4; F3-C3/F7-T3; F3-C3/T3-T5; F3-C3/Fp2-F8; C3-P3/T3-T5; P3-O1/T5-O1; Fp2-F4/Fp1-F7; Fp2-F4/F7-T3; Fp2-F4/Fp2-F8; Fp2-F4/F8-T4; F4-C4/C4-P4; C4-P4/T4-T6; C4-P4/Cz-Pz; P4-O2/T6-O2; Fp1-F7/F7-T3; Fp1-F7/Fp2-F8; Fp1-F7/F8-T4; F7-T3/Fp2-F8; Fp2-F8/F8-T4
Edges common to both masks	Fp1-F3/Fp1-F7; F3-C3/F7-T3; C3-P3/T3-T5; P3-O1/T5-O1; C4-P4/T4-T6; C4-P4/Cz-Pz; P4-O2/T6-O2

ASD edges were significantly below the surrogate ASD bootstrap distribution, and control edges were significantly above the surrogate control bootstrap distribution

### Data preprocessing for network and spectral analysis

For network analysis, the EEG data were filtered with a 3^rd^ order Butterworth, zero-phase filter (notch filtered at 60 Hz to remove line noise, high pass at 0.5 Hz to avoid slow drift, and low pass at 50 Hz to avoid higher-frequency line noise harmonics). Because the EEG data were selected to avoid large movements and muscle artifact, noncontiguous points occurred; we removed 0.5 s from both sides of each noncontiguous point before further analysis. Visual analysis and a simulation study (not shown) confirmed that this removal was sufficient to mitigate artifacts produced at the noncontiguous points during the filtering process. For spectral analysis, the EEG data were not filtered, but 0.5 s was removed from each noncontiguous point to maintain consistency with the network analysis. In order to optimize near-field activity and reduce electrical contamination from the physical reference, both filtered and non-filtered data were then re-referenced according to the longitudinal bipolar (‘double banana’) montage, leaving 18 bipolar signals (‘derivations’) in place of the original 19 electrode signals. This reference montage was chosen in lieu of other popular montages such as the common average or Hjorth-Laplacian references because of its effectiveness and widespread clinical usage. While the common average reference and spline Laplacian reference perform reasonably well when used with a large enough number of electrodes (e.g., 128 or more), these references are expected to perform poorly when applied to the standard, low density 10/20 electrode system (see [54], page 295). In addition, the common average reference has been found to increase spurious coupling in some cases [55]. In contrast, bipolar montages are considered one of the best available options to improve spatial resolution in EEG with a limited number of electrodes (see [54], p. 291). Hjorth (or nearest-neighbor) Laplacian is closely related, however we chose the double banana montage due to its extensive use clinically.

All EEG data were then divided into non-overlapping windows of 2 s duration (windows containing concatenated data from noncontiguous time points were discarded). We use 2 s intervals to approximately maintain stationarity in the time series (which requires short epochs) while keeping sufficient data for accurate coupling estimates (which requires long epochs). Finally, we normalized the data from each electrode within each window to have zero mean. All data preprocessing and subsequent analysis were performed using custom software developed in MATLAB.

### Spectral analysis procedure

For the spectral analysis of the unfiltered data, the power spectrum for each 2 s epoch was computed using the multitaper methods implemented in the Chronux toolbox [56] with 5 tapers and a time-bandwidth product of 3, so that the resulting frequency resolution was 1.5 Hz. Frequencies below 0.5 Hz were omitted to avoid low-frequency drift in the data. For each subject this resulted in a power spectrum for each of the 18 re-referenced signals, for each 2 s epoch.

**Fig. 1:**
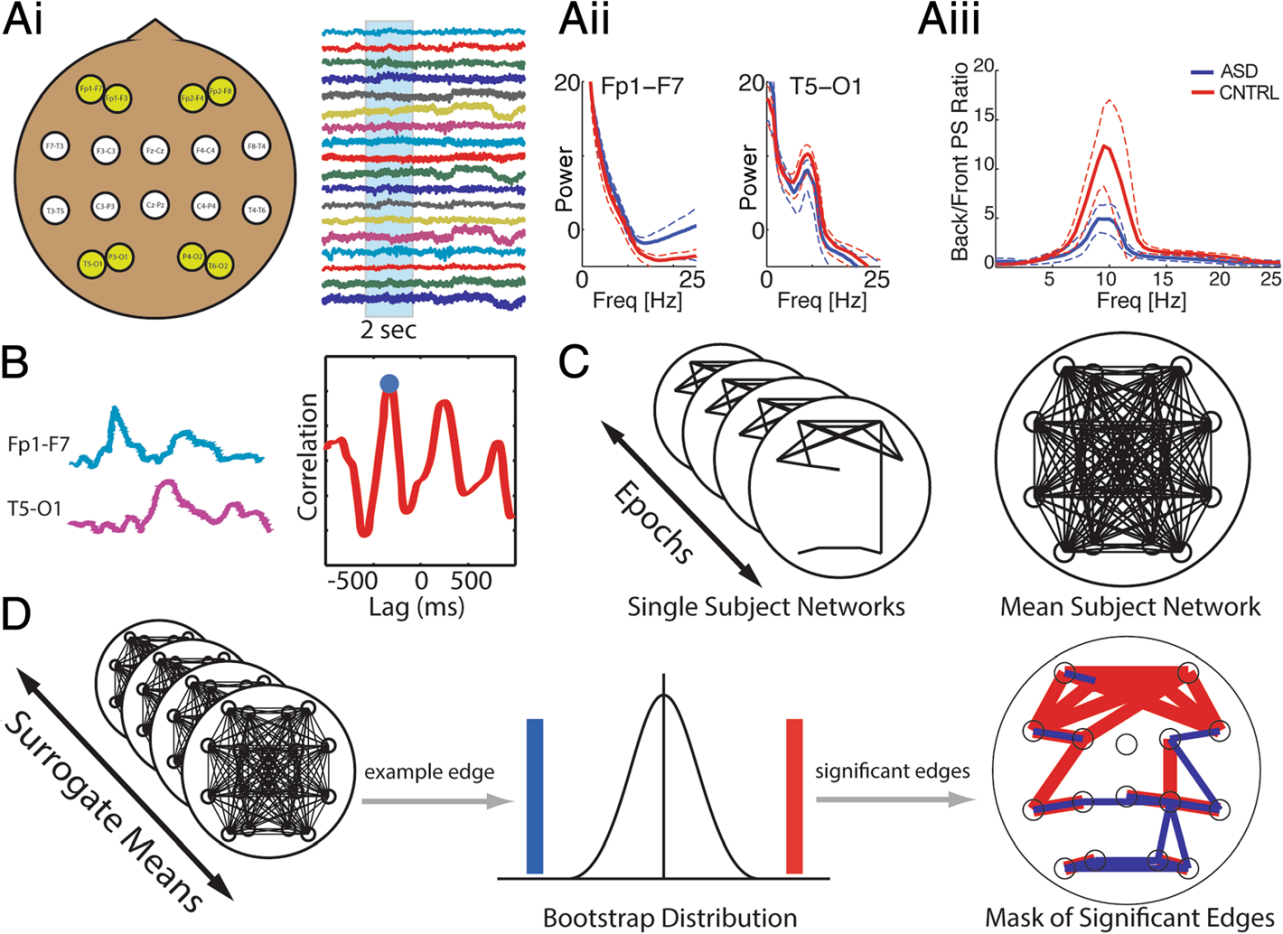
Construction of power ratio and functional networks from multivariate scalp EEG recordings. **ai** Example EEG data from re-referenced 18 channels (broadband, 0.5 - 50 Hz) according to the bipolar “double banana” montage. Filtered and unfiltered data are divided into 2 s epochs. **aii** From unfiltered data power spectra are calculated for each channel using the multitaper method. **aiii** The ratio of power spectra are obtained from the power spectra of the posterior four derivations (T5-O1, P3-O1, P4-O2, T6-O2) divided by the anterior four derivations (Fp1-F7, Fp1-F3, Fp2-F4, Fp2-F8). Shown here is the mean posterior/frontal power spectra ratio to illustrate the properties of the peak alpha-ratio statistic. **b** For each channel pair filtered data (0.5 - 50 Hz) from 2 s epochs are used to calculate the cross-correlation. Two example traces for Fp2-F8 and T4-T6 show a correlation here with maximal coupling at a time lag of −50 ms. The significance of the maximum absolute value of the cross-correlation (blue circle) is determined using an analytic procedure (see Methods). **c** Example binary coupling networks derived from four 2-s epochs. Significant electrode coupling is represented with an edge. These networks are averaged, resulting in a weighted coupling network for each subject. These are then compared against bootstrapped edge weight distributions in (**d**). **d** To create bootstrapped edge weight distributions, surrogate networks mirroring the original datasets are created by randomly sampling functional networks with replacement from all epochs of all subjects of both groups. Original ASD and control edge weights are compared to the surrogate edge weight distributions, and edges most significantly outside the distribution (*p* < 1/100,000) are selected to make a mask of highly significant edges. This mask is used to select the edges with the greatest difference between the ASD and control groups

To characterize the power spectra for each patient we computed a summary statistic – the “peak alpha-ratio” – as follows (Fig. 1). First, we computed the power spectrum of each signal for each epoch of the dataset, and then averaged the power spectra across all epochs. Second, we computed the ratio of this average power between four pairs of posterior to anterior signals (Far Left: T5-O1/Fp1-F7; Medial Left: P3-O1/Fp1-F3; Medial Right: P4-O2/Fp2-F4; Far Right: T6-O2/Fp2-F8). Third, we determined the maximum value of the ratio within the alpha frequency range (8–14 Hz) for each of the four channel pairs. These four maximum back/front ratios were then averaged to produce the summary statistic, mean “peak alpha-ratio”, for each patient. We choose to compute the spectral ratio for three reasons. First, the posterior to anterior alpha gradient is one of the most widely observed EEG features in healthy controls and thus is an intuitive feature to evaluate in a disease population [57]. In addition, this metric has been previously correlated with behavioral inhibition and sociability [58, 59]. Second, as described in Results, changes in power (not the ratio) between the ASD and control subjects at all electrode deviations reveal no significant differences. Third, we choose to compute the frontal/posterior ratio to normalize the spectral results of each individual subject. This choice of normalization protects against artifacts that impact the overall amplitude of voltage activity for each subject (e.g., a subject with thicker hair may be expected to have reduced electrode conductance and an overall reduction in EEG amplitude), and we expect this normalization to make the results more robust to changes in clinical settings and routine (e.g., to changes in electrode recording equipment).

### Functional network inference and measures

While there are many approaches to determining functional connectivity from time series data [60], including multiple coupling measures (e.g., linear or non-linear) and different strategies for determining network edges, we selected a simple measure of linear coupling: the cross correlation. The cross correlation is a bivariate measure of linear association between two brain regions, and serves as a basic measure of electrocortical functional connectivity [24, 61]. We note that most linear and nonlinear measures appear to perform equally well on simulated and observed macroscopic brain voltage data [62, 63].

Each subject possessed at least 50, 2-s epochs of data (min 57, max 1256, mean 254), which is sufficient to support stable functional network representations [64–66]. To create functional networks, we follow the procedure outlined in [67] and applied in [64–66]. We briefly describe this procedure here (Fig. 1). For each patient, we create a functional network for each 2 s epoch of filtered data using the 18 derivations (signals) of data, based on the cross correlation of the data between each pair of derivations. We note that each signal in each 2 s interval is normalized by its variance (or total power) before performing the correlation analysis. Doing so reduces the differences in amplitude between signals and mitigates a potential confounding factor in the correlation analysis [68]. In addition, we show in Results that differences in correlation between the ASD and control subjects are not accompanied by changes in the (absolute) EEG power in the 2.5-17.5 Hz range (i.e., the broad, low frequency range which dominates the correlation measure). This observation suggests that changes in EEG power (i.e., in the signal to noise ratio) do not confound the functional connectivity results, in accordance with [68, 69]. We use the maximum absolute value of the cross correlation over time lags of ±500 ms to measure the coupling (which encompasses the duration of known neurophysiological processes and cross-cortical conduction times [70, 71]). To assess the variability of the cross correlation across lags, we compute the average variance of the cross correlation between all derivation pairs and all 2 s epochs for a subject; this provides a common measure of variability that we apply to assess the significance of each correlation statistic (see [67]).

For each 2 s epoch, an undirected binary functional network is inferred from these correlations based on their significance. Each node represents a derivation (e.g., channel T5 – channel O1), an edge value of 1 represents a statistically significant correlation between the two derivations, and an edge value of 0 indicates a weaker correlation. To correct for the multiple significance tests within each 2 s epoch, we use a linear step-up procedure controlling the false detection rate (FDR) with *q* = 0.05. For this choice of q, 5 % of the network connections are expected to be falsely declared [72]. This procedure results in a thresholding of the significance tests of the correlation — not of the correlation value itself — for each 2 s epoch [67]. The networks obtained in this manner have an associated measure of uncertainty, which is the expected number of edges incorrectly declared present.

To mitigate the impact of volume conduction [54, 61] on the functional network analysis, we identified the correlations deemed significant at zero lag, and removed these edges from the analysis. In doing so, we expect to remove both spurious correlations due to volume conduction and true correlations that occur at zero lag; in this sense, this procedure is conservative. This approach has an added benefit of reducing the effect of montage selection, whereby subtraction of signals may result in spurious coupling between derivations that share electrodes.

To assess the network structure, we apply two measures of network connectivity [23]. The density for each network is calculated in the standard way as the number of edges detected (at non-zero lag) divided by the total number of possible edges (153 minus the number of spurious edges detected at zero lag). The mean density for each subject is calculated as the average density across all epochs for the subject. The mean density for each group (ASD and control) is calculated as the mean of the subject densities within each group. The degree is also calculated in the standard way as the number of edges that connect to each node, and average degree values for a subject and group are calculated in the same way as the average density values.

In addition to correlation networks, we also computed networks with a second measure of linear association - the coherence, estimated using the multi-taper method [73]. As for the correlation networks, we inferred coherence networks for all derivative pairs over 2 s epochs. To calculate a p-value to identify significant edges in the coherence networks, we first transformed the coherence, C, to the quantity (ν_0_ − 1)|C|^2^/(1 − |C|^2^), which has an approximate F-distribution with two and ν_0_ − 2 degrees of freedom under the null hypothesis of no coherence. Here, ν_0_ is twice the number of tapers, either 10 or 16. We then corrected for multiple significance tests using a linear step-up FDR controlling procedure with *q* = 0.05. Coherence networks were computed for four electrode montages - double banana, transverse, Hjorth Laplacian, and neck reference – and for both sleep and wake data, at 4 frequencies with 5 Hz bandwidth and 8 tapers (centers at 3.5 Hz, 8.5 Hz, 13.5 Hz, and 18.5 Hz) and 8 frequencies with 3 Hz bandwidth and 5 tapers (centers at 2.5 Hz, 5.5 Hz, 8.5 Hz, 11.5 Hz, 14.5 Hz, 17.5 Hz, 20.5 Hz, and 23.5 Hz). However, we found no significant differences in density between the ASD and control groups in the exploratory analysis, and the analysis of coherence networks was not continued in the validation dataset.

### Bootstrap test for significantly different edges

With the aim of developing a biomarker for ASD, we sought to assess the difference in network structure between the ASD and control groups. While a network-wide measurement such as the density is informative, a measure that localizes differences between ASD and control networks to more specific connections (e.g., network edges) would provide additional information. Knowledge of specific edge differences would allow us to focus on just these edges, reducing noise introduced by non-informative edges, and potentially producing a more sensitive and specific biomarker.

To that end, a bootstrap analysis was performed to test whether a significant difference occurs between the ASD and control groups in the appearance of each edge. We began with the null hypothesis that no difference exists between the two populations. We then created surrogate data for each subject by randomly drawing with replacement functional networks (each derived from a 2 s epoch) from the combination of all ASD and control subjects. This process of generating surrogate data was then repeated for all subjects. In this way, the surrogate data for each subject of each group was created. If the null hypothesis is correct, we should find no statistically significant differences between the network features deduced from the original ASD and control groups compared to the surrogate data.

We repeated this process of generating surrogate data and computing network measures 100,000 times to create a distribution of average edge weights for each edge in the ASD group and in the control group. For each of the 100,000 surrogates of both groups, 153 average edge weights were calculated (one for each node pair). We note that the average edge weights were calculated in the same way as for the original data; that is, for each subject we computed an average network across the 2 s epochs, and then these subject networks were averaged to produce a population average network for the ASD group, and a population average network for the control group. The 100,000 surrogates correspond to 100,000 population average networks for the ASD group, and 100,000 population average networks for the control group. In these surrogate data, the 100,000 values for each edge weight establish the bootstrap distributions of the edge weights for the ASD group and control group.

We then compared each observed average ASD edge weight to the corresponding surrogate ASD distribution, and each observed average control edge weight to the corresponding surrogate control distribution. This bootstrapping allows us to examine each edge individually, and to determine the statistical significance of particular edges in the ASD and control groups. Finally, we determined the subset of edges identified as the most significantly different in the observed data compared to the surrogate data. In practice, these edges were associated with the smallest p-values detectable in the bootstrap procedure (*p* < 10^−5^). The edges identified in this way were used to generate a “mask”, or selection of edges most significantly different from the bootstrap distribution, with the purpose of developing a biomarker of ASD (Fig. 1).

### Classification of datasets

We also performed a discriminant analysis to classify the validation subjects into ASD and control groups. To do so, we used the MATLAB function *classify*, selecting the classification option ‘quadratic’; in this method, a discriminant function fits a multivariate normal density to each group, with covariance estimates stratified by group. We trained the classifier on the first group of ASD and control subjects, and tested the classifier on the second group of validation subjects using the two validated significant quantitative measures identified in the training data (peak alpha ratio and mask density, i.e., the mean weight of a subset of edges).

## Results

In this section, we describe the application of spectral and network analysis to EEG data recorded from ASD and control subjects. Using an initial exploratory analysis on a subset of ASD and control subjects, we build hypotheses that we then test in a second group of ASD and control subjects. In this way, we identify and validate two biomarkers of ASD that can be inferred from standard EEG clinical recordings. Below we briefly review the exploratory measures tested and then describe in detail the measures tested on the validation dataset. In the spectral analysis, we focus on a measure of the antero-posterior spectral power gradient, a metric previously correlated with behavioral inhibition and sociability [58, 59]. In the network analysis, we first examine the network density. We then examine node degree to determine if the differences in density are driven by a subset of spatial locations, or are a property of the entire network. We next identify a subset of edges that appear significantly more common in the control group, and significantly less common in the ASD group. These select edges provide a subset of highly significant edges to apply in a biomarker. Finally, we attempt to classify the two populations in the validation group using a subset of measures deduced from the training group.

### Spectral analysis reveals an alpha-band biomarker of ASD

**Fig. 2:**
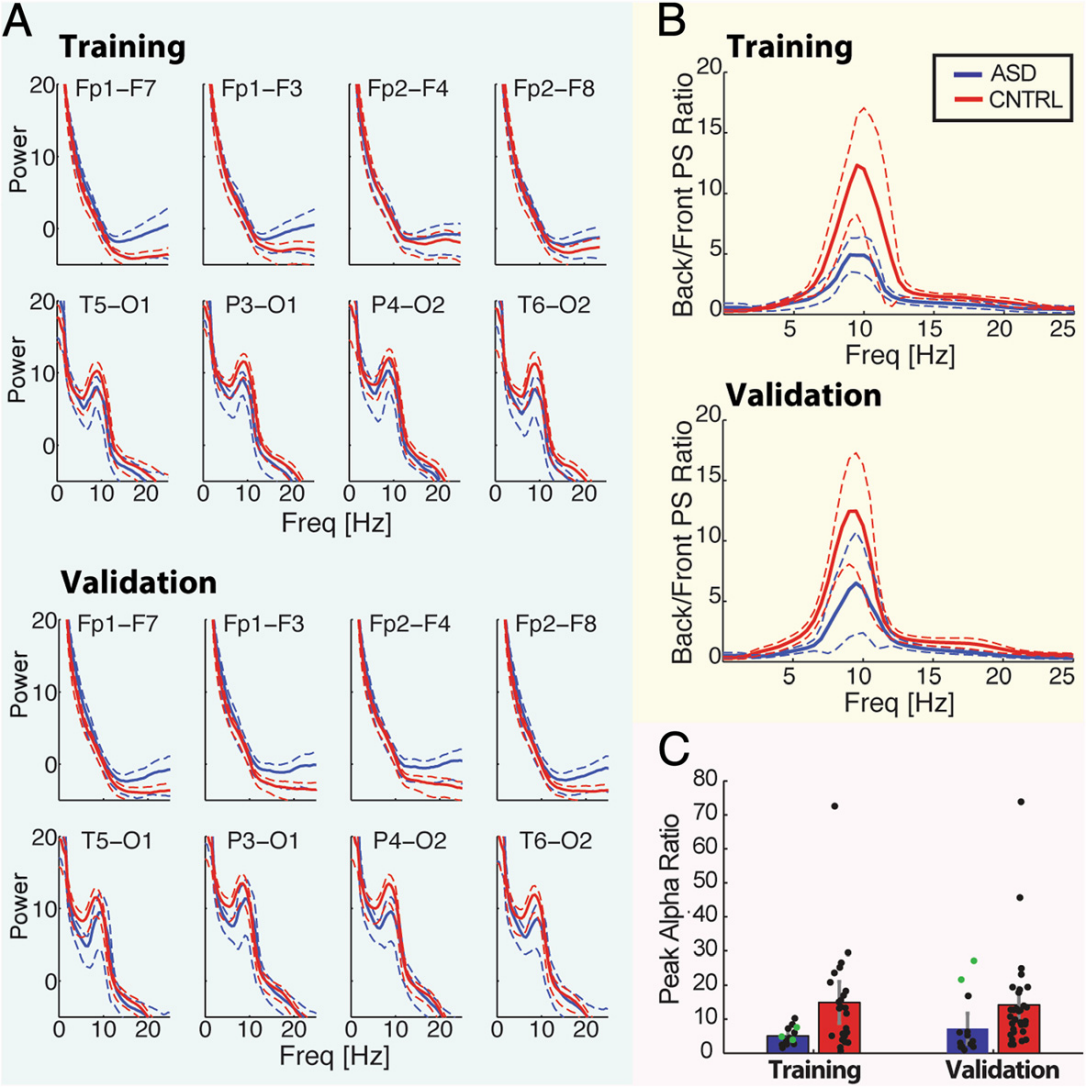
Posterior to anterior power spectra ratio differs significantly between ASD and control groups. **a** Power spectra of ASD and control groups recorded at the anterior (Fp1-F7, Fp1-F3, Fp3-F4, Fp2-F8) and posterior (T5-O1, P3-O1, P4-O2, T6-O2) nodes, calculated for each 2 s epoch, and averaged over all epochs. Training group analysis (top) and validation group analysis (bottom). Dashed lines represent two standard errors of mean. Power in units of 10log_10_ (μV^2^/Hz). **b** Averaged power spectra ratio between posterior and anterior channels (i.e., T5-O1/Fp1-F7) averaged over epochs and computed at four locations, then averaged over subjects to create a group average for the training (top) and validation (bottom) groups. Upper and lower 95 % confidence bounds indicated by dotted blue (ASD) and dotted red (control) lines. This result motivated the creation of the peak alpha-ratio statistic (Fig. 2c). **c** For each epoch, the maximum values were obtained of the four power ratios, in the alpha frequency band, and averaged. These ratios were then averaged over all epochs for each subject in the ASD (blue, Asperger’s in green) and control (red) groups. The peak alpha ratio is lower in the ASD group in the training (p ≤ 0.0034) and validation data (*p* ≤ 0.0025). Error bars represent two standard errors of the mean

To assess rhythmic activity in the EEG data, power spectra were computed from numerous short epochs (Fig. 2, also see Methods: Spectral analysis procedure). Visual inspection of the average power spectra during wakefulness suggests differences between the ASD and control groups (Fig. 2a, top two rows): the anterior power spectra have higher mean power in the ASD subjects (blue) than the control subjects (red) at alpha frequency and above (plateauing near 20 Hz). In addition, visual inspection suggests that both ASD (blue) and control (red) subject population mean power spectra possess a broad peak in the alpha frequency range (~10 Hz) in the posterior four channels, consistent with the well characterized posterior dominant alpha rhythm present in quiet wakefulness [74, 75]. We note that visual inspection of the power spectra of the posterior four derivations reveals a larger peak in alpha power of the control subjects (Fig. 2a, red) compared to the ASD subjects (Fig. 2a, blue).

To characterize these differences in power, for each subject in the exploratory group we calculated the mean power from 2.5 Hz to 17.5 Hz, in steps of 3 Hz. We note that this analysis covers frequencies across the classically defined frequency bands. We omitted higher frequencies due to the increased relative impact of muscle artifact in those ranges [76]. Using this metric, over these 6 frequencies and 18 derivations, we found 9 significantly different power values between the ASD and control populations out of 108 comparisons (significances ranged from *p* = 0.0099 to 0.0459). When corrected for multiple comparisons using a False Detection Rate (FDR) control, no significant differences remained. We also computed power spectra for NREM sleep data in the exploratory group, but found by visual inspection that the mean power spectra for the ASD and control populations at each derivation overlapped. We therefore did not explore the sleep data further.

Because no significant differences were found at individual derivations, we considered ratios of power. Absolute differences in mean power are expected to be muted by our normalization procedures, however power ratios will not be affected. Population differences in the anterior and posterior derivations are enhanced in the averaged posterior/frontal spectral ratios (Fig. 2b). Visual inspection of the spectral ratio of posterior derivations to frontal derivations averaged across derivations and subjects revealed a peak most prominent in the alpha band. In the exploratory group, the ratios exhibit a sharp peak near 10 Hz for both control (Fig. 2b, red) and ASD (Fig. 2b, blue) subjects, and the peak is visibly larger in the control group compared to the ASD. To further characterize these results beyond visual inspection, we calculate a single measure, here labeled the “peak alpha ratio”. This measure focuses on the antero-posterior EEG spectral power gradient, and provides a numeric summary statistic to compare the ASD and control groups. The peak alpha ratio is calculated for each subject at every 2 s epoch as follows (for detailed description, see Methods: Spectral analysis procedure). Power spectra are averaged over all epochs, and then the four posterior derivations are divided by the corresponding power spectra of the four anterior derivations, which results in four spectral ratios as a function of frequency (from 0 to 60 Hz). The maximum value in the alpha frequency (8–14 Hz) band of each ratio is determined, and then the four maximal values are averaged to produce a single alpha ratio for the epoch. Alpha ratios for all epochs for a subject are then averaged to produce a mean alpha ratio for the subject (Fig. 2c). Applying this measure to the exploratory group, we find that the ASD population has a significantly lower mean peak alpha ratio (*p* ≤ 0.0034) than the control population, consistent with the visual inspection results (Fig. 2a,b). We note that the peak alpha ratios for EEG data recorded during sleep in the exploratory group showed little to no peak, and ASD and control values exhibited no significant differences.

Spectral features in the second group of subjects – the validation group – remain much the same (Fig. 2). In the validation group, the ASD population’s mean peak alpha ratio is significantly below that of the control population (*p* ≤ 0.0025). We conclude that the significant spectral features identified in the first group of subjects are validated in the second group of subjects.

### Functional network analysis reveals specific biomarkers of ASD

#### Network density

**Fig. 3:**
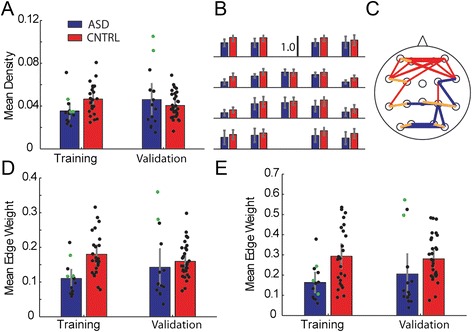
Network analysis reveals that select edges show a significantly diminished density in ASD versus control groups, though not in overall mean density. **a** Mean density of ASD (blue, Asperger’s in green) and control (red) groups. In the training data, the mean density of the ASD group was significantly lower than the mean density of the control group (p ≤ 0.028). However, in the validation data no significant difference was found (*p* = 0.50). Error bars represent two standard errors of the mean. **b** In the training data, no significant difference in degree between ASD and control groups was found at any node location. **c** The “edge mask”. Edges in the mean control network which were significantly greater than the surrogate control distribution are shown in red (*n* = 23), while edges in the mean ASD network which were significantly lower than the surrogate ASD distribution are shown in blue (*n* = 16). Seven edges (shown in orange) were found to distinguish both control from surrogate and ASD from surrogate, and were retrospectively used to form a mask of highly selective edges. **d** The mask density reveals a significant difference between the ASD group and the control group in training data, as expected (*p* ≤ 0.0019). The mask density of the ASD group was significantly lower than the mask density of the control group (*p* ≤ 0.0085) in the validation data as well. **e** In a retrospective study, the intersection mask density was computed. In both the training and validation data, the ASD intersection mask density was found to be significantly lower than the control intersection mask density (*p* ≤ 0.0163 in training data, *p* ≤ 0.0006 in validation data)

After inferring the functional networks from the EEG data (see Methods, Functional network inference and measures), we investigate differences in network topology between the ASD and control groups. Many statistics exist to assess network structure [13, 23]; here we focus on one of the most fundamental – the density – which is computed by summing the number of edges in a network, and then dividing by the number of possible edges. We note that, for the functional networks inferred here, a higher density value indicates an increased level of correlation within the network. The mean density across epochs was calculated for each subject, and averaged within-group (Fig. 3a). In the training group, the ASD population produced a significantly lower mean density than that of the control population (*p* ≤ 0.028), consistent with some findings in the literature [16, 18, 40, 77–81]. However, in the validation analysis, we found no significant difference in density between the two groups (*p* = 0.502, Fig. 3a).

We also examined the mean density for EEG data recorded during NREM sleep in the exploratory group, but we detected very few edges (approximately 0.005 density) and found no significant difference between the mean ASD and control populations (*p* = 0.4578). Finally, we investigated the density in the correlation networks using alternative reference montages, including the transverse and Hjorth Laplacian [54] reference montages. The results were similar to the double banana montage for the wake data (Additional file 1: Figure S1) and for the sleep data. In addition to analysis of correlation networks, we also considered networks inferred using a frequency domain measure of linear association: the coherence (see Methods). We computed the density of these coherence networks for the exploratory group sleep and wake data using four reference montages (double banana, transverse, Hjorth Laplacian, and neck) at 4 frequencies with 5 Hz bandwidth (centers at 3.5 Hz, 8.5 Hz, 13.5 Hz, and 18.5 Hz) and 8 frequencies with 3 Hz bandwidth (centers at 2.5 Hz, 5.5 Hz, 8.5 Hz, 11.5 Hz, 14.5 Hz, 17.5 Hz, 20.5 Hz, and 23.5 Hz). Using the double banana montage on wake data, at 5 Hz bandwidth, the coherence densities were found to have no significant difference except at 13.5 Hz (*p* = 0.0055). At 3 Hz bandwidth, the only significant difference between ASD and control was found at 14.5 Hz (*p* = 0.0312). At this frequency the coherence density values were low (approximately 0.01), and further examination revealed that the significance was driven by two control subjects with high coherence values, thus this result was not considered reliable. The coherence results were not examined further in this study.

In an effort to spatially localize the functional network differences between the ASD and control populations, we computed measures of localized density of different brain regions between groups. To compute these measures we restricted the derivations used in our density calculation to a subset of the network corresponding to a specific region of the scalp, comparing the left and right hemispheres, and the anterior to posterior halves of the network. We found the ASD population mean density to be significantly lower than the control population mean density in the left hemisphere (*p* = 0.036), right hemisphere (*p* = 0.013), and near significance in the anterior hemisphere (*p* = 0.073) and posterior hemisphere (*p* = 0.058). The left-right inter-hemispheric density (i.e., the density of connections between left and right hemispheres) difference was also nearly significant (*p* = 0.058), but the anterior-posterior inter-hemispheric density (i.e., the density of connections between nodes in the anterior and posterior halves of the full network) was not found to differ significantly between the groups (*p* = 0.954). These results indicated that density differences were not localized to specific regions, but were distributed throughout the brain. Because the overall change in density was not validated in the second data group, and because network degree analysis likewise did not indicate localization of differences in connectivity (see Network degree below), we did not perform analysis of the density in specific brain regions in the validation population.

#### Network degree

In an effort to spatially localize the functional network differences between the ASD and control populations, we computed a second statistic – the degree – which measures the number of edges that contact a node. In the training data (Fig. 3b, top) we tested each node between ASD and control populations and found no significant differences when Bonferroni corrected (*p* > 0.0028 for all edges, where Bonferroni correction of *p* = 0.05 for 18 comparisons is 0.05/18 = 0.0028), indicating no evidence for spatial organization of the difference in the node degree between the two groups.

#### Mask density

**Fig. 4:**
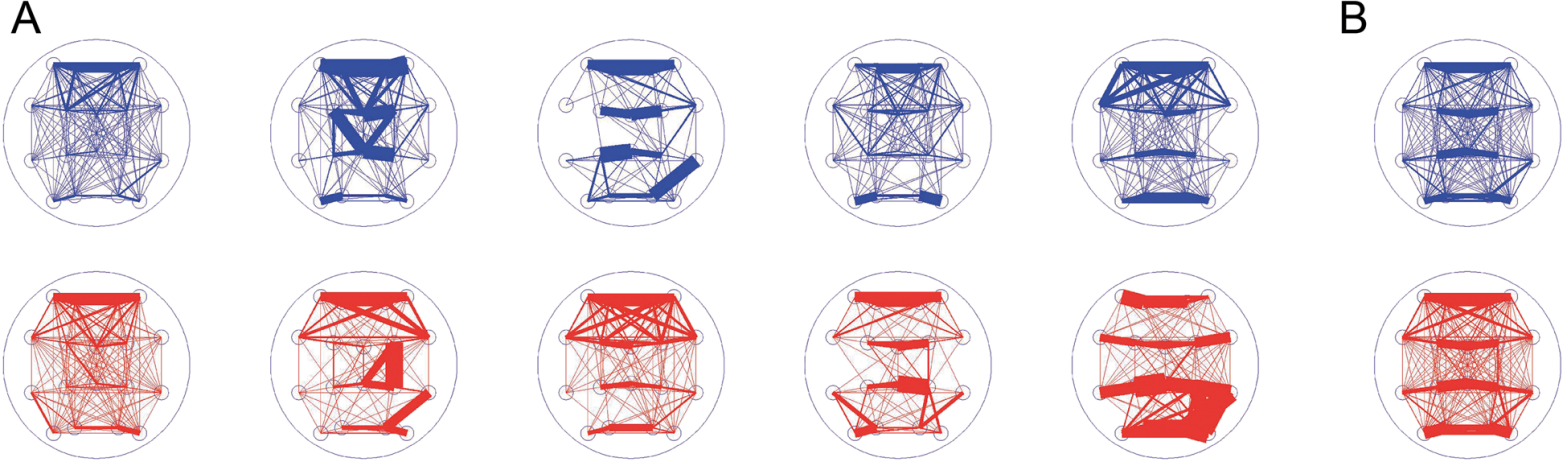
Sample mean networks for the ASD and control subjects exhibit variability, and the mean group networks exhibit qualitatively similar patterns. **a** Example networks from 5 ASD subjects (top row, blue) and 5 control subjects (bottom row, red) are shown to demonstrate how individual subjects varied in their mean network edge weights. While some edges were consistently more represented (as in the frontal area, for example), individual subjects did not exhibit identical network weight patterns across the group. **b** Mean group networks for ASD (top row, blue) and control (bottom row, red) appear to have superficially similar patterns of edge weights

We expect high variability in the functional networks inferred from each 2 s epoch, as the brain responds to evolving internal and external demands. To establish more stable functional network representations, we computed the average functional network of each patient. In practice, the average functional network is the mean of all functional networks inferred across time for a patient. The average functional network is a weighted network, in which the edge weight indicates the proportion of times that edge appears in all epochs for a patient. For example, an edge weight of 0 indicates that two pairs of sensors (i.e., derivations) are never correlated across all 2 s epochs, while an edge weight of 1 indicates two pairs of sensors that remain correlated in each 2 s epoch. We have recently shown that average functional networks computed for more than 100 s of data constitute stable network templates or “cores” [64, 66]. These *template networks* computed for the ASD and control subjects reveal heterogeneous network structures within each group, rather than a common difference visually distinguishing each ASD subject from each control subject (Fig. 4a). We then computed the mean of these template networks across subjects within each group, resulting in the mean ASD template network, and the mean control template network (Fig. 4b). The mean ASD and control template networks displayed grossly similar structures, with slight differences in specific edge weights difficult to discern from visual inspection alone.

We next considered whether these template networks facilitate the development of an additional biomarker of ASD. To do so, we investigated specific edge weights. To identify those edges that differed most significantly between the ASD and control subjects, we generated surrogate network data. Briefly, we generated these surrogate data under the null hypothesis of no difference between the ASD and control populations (see Methods, Bootstrap test for significantly different edges). We find in the mean ASD template network 16 edges with significantly lower weights than in the surrogate ASD distribution (*p* ≤ 10^−5^), and two edges with significantly greater weights than in the ASD surrogate distribution (*p* ≤ 10^−5^). Conversely, we find in the control population 23 edges with significantly higher weights than in the surrogate control distribution (*p* ≤10^−5^), and only one edge with significantly lower weight than in the surrogate control distribution (*p* ≤ 10^−5^). Using the 16 edges from the ASD population significantly lower than their surrogate distribution and the 23 edges from the control population significantly higher than their surrogate distribution we constructed an *edge mask* (Fig. 3c), representing a candidate subset of edges to distinguish the ASD and control groups (Table 2). If these edges are truly selective, then analysis focused only on these edges should improve the distinguishability of the ASD and control populations beyond a global network density measure that includes all edges.

To quantify this in a summary statistic, we computed the proportion of edges in the edge mask for each network in all subjects and both populations. The result is a single statistic for each network, which we call the *mask density*. For each subject, we compute the average mask density across all 2 s epochs. In the training analysis, we find a significantly higher mask density in the control group versus the ASD group (*p* ≤ 0.0019, Fig. 3d). We then applied the same edge mask – deduced from the training data - to the validation data. Here, we again find that the mask density is significantly higher in the control networks compared to the ASD networks (*p* ≤ 0.0085, Fig. 3d). In this case, the connectivity strength of a subset of edges serves as a robust biomarker of ASD.

### Post-hoc measures

To further explore features of the population data, we performed the following eight analyses after validation of the two biomarkers.

#### Classification model

**Fig. 5:**
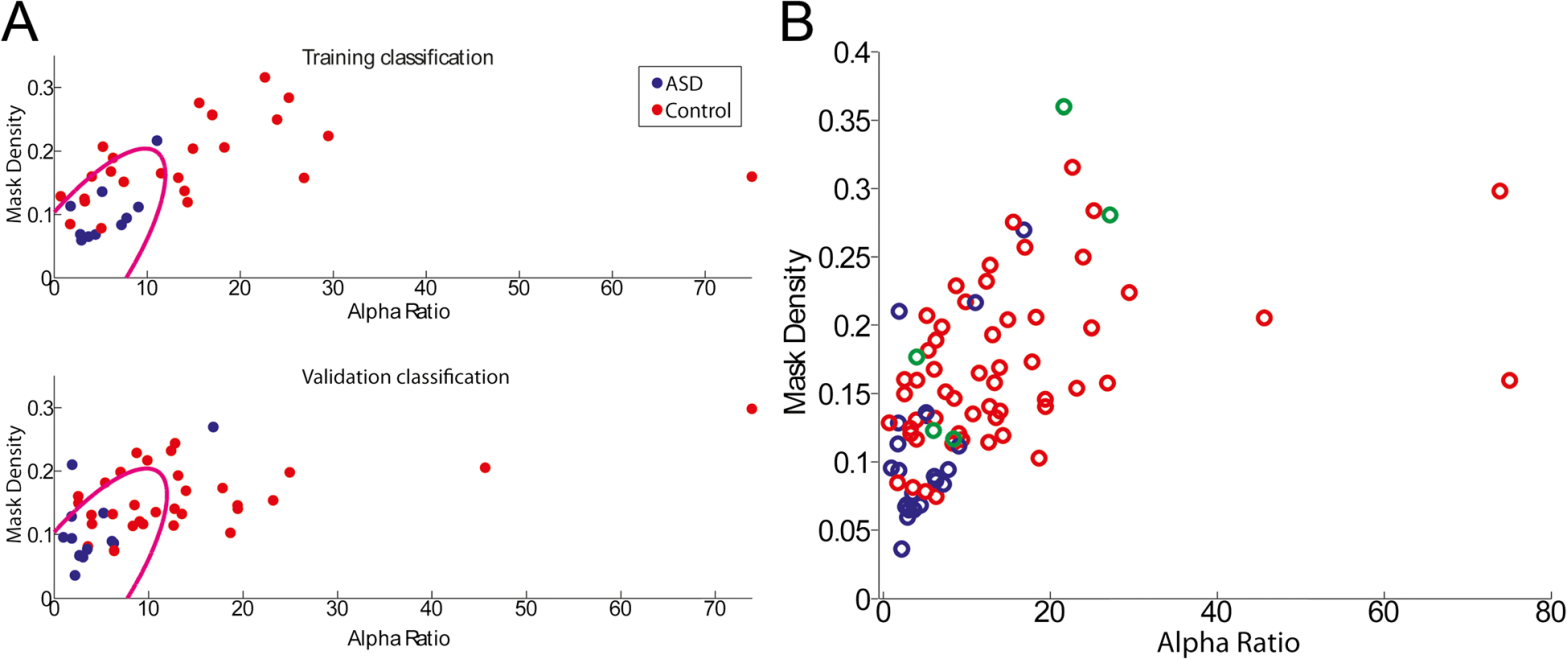
Discriminant analysis classifies the ASD and control groups. **a** Scatter plots of the quadratic discriminant analysis (QDA) using the two validated measures of peak alpha ratio and mask density. Classification rate was 83 % sensitivity and 68 % specificity. For classification purposes, Asperger’s subjects were excluded. The quadratic classification curve (magenta) was trained on the training data population (top) and used to classify the validation data population (bottom). **b** The validated measures for all patients (both the training and validation groups), and including the Asperger’s subjects (green circles), displayed here for visualization. Visual inspection suggests a difference between ASD and control populations, and that Asperger’s subjects are removed from the main cluster of ASD subjects

We performed a quadratic discriminant analysis (QDA) classification with the two validated biomarkers. Using the peak alpha ratio and the mask density, we trained a QDA classifier on the training group of subjects (excluding Asperger’s subjects), and then tested this classifier on the validation group of subjects (excluding Asperger’s subjects). The QDA classifier successfully classified ASD subjects with 83 % sensitivity (10/12 correctly classified as ASD) and 68 % specificity (21/31 subjects correctly classified as control, Fig. 5).

#### Edge mask

The mask used in the initial classification was a union of edges from the ASD mean network that were significantly less than surrogate distribution, and edges from the control mean network that were significantly greater than surrogate distribution. There were seven edges that were common to these two groups, which we call the *intersection mask*. To investigate whether this intersection mask better discriminated the ASD and control groups, we performed a post-hoc calculation of this intersection mask density (using only the seven edges identified, see Table 2) and classification analysis similar to that described above, but now using the intersection mask. Consistent with the results reported above, we find a significantly higher intersection mask density in the control group versus the ASD group in the training data (*p* ≤ 0.0163, Fig. 3e) and in the validation data (*p* ≤ 0.0006, Fig. 3e). Using the peak alpha ratio and the intersection mask density, we then trained a QDA classifier on the training group of subjects, and then tested this classifier on the validation group of subjects. The QDA classifier performed similarly to the classification with the union mask, and successfully classified ASD subjects with 83 % accuracy and 64.5 % specificity.

#### Gender

ASD is known to be more common in males than females [82–84]. We performed post-hoc analysis of male versus female subjects using control subject data only, and found no difference in density or mask density. For the spectral analysis, there was a significantly higher peak alpha ratio found for male subjects compared to female subjects in the training group (20.46 versus 8.38, *p* = 0.039), such that the peak alpha ratio in neurotypical females was closer to ASD values; the peak alpha ratio was also greater in males compared to females in the validation group, however this difference was not significant (14.33 versus 14.10, *p* = 0.3792).

#### Asperger’s syndrome

Five of our ASD subjects met criteria for Asperger’s syndrome (a less severe form of ASD [85]), three from the exploratory population and two from the validation population. When evaluated separately, we found that the results for the Asperger’s subjects were either between the ASD-only and the control subject averages or outlying to both overall populations (see green markers in Figs. 2, 3, 5). For the exploratory population, the alpha ratio was 5.5 for ASD subjects excluding Asperger’s subjects, 6.2 for Asperger’s subjects, and 15.2 for control subjects; while mean density was 0.034 for ASD subjects excluding Asperger’s subjects, 0.039 for Asperger’s subjects, and 0.046 for control subjects; the mask density was 0.10 for ASD subjects excluding Asperger’s subjects, 0.14 for Asperger’s subjects, and 0.18 for control subjects. For the validation population, the alpha ratio was 4.31 for ASD subjects without Asperger’s subjects, 24.40 for Asperger’s subjects, and 14.20 for control subjects, while mean density was 0.037 for ASD subjects without Asperger’s subjects, 0.099 for Asperger’s subjects, and 0.041 for control subjects, and mask density was 0.113 for ASD subjects without Asperger’s subjects, 0.320 for Asperger’s subjects, and 0.160 for control subjects. Inclusion or exclusion of the Asperger’s subjects in the ASD group altered the p-values reported above, did not affect the significance of the results reported. These observations for a small group (five) of Asperger’s subjects suggest further study from a larger population is warranted.

#### Data stability

In the exploratory group we examined the effects of differences of number of epochs, number of subjects in each population, and differences in number of subjects at each age for the density and peak alpha ratio. We compared ASD and control populations using the first 100 epochs, the last 100 epochs, 100 randomly selected epochs, and in all cases found the ASD density to be consistently significantly lower than control population mean density. We also repeated this same analysis using only 13 control subjects (to equal the number of ASD subjects), randomly selected 100 times, and in each case found changes in density and peak alpha ratio consistent with the previous analysis. Finally, we repeated the analysis above, but first averaged subjects within each age group, and then across the age groups, and found results consistent with the previous analysis, both for density and for peak alpha ratio.

#### Effects of the presence of sleep

To evaluate whether sleep, or lack thereof, impacted group differences on the wake EEG, we conducted a post-hoc analysis of the two validated biomarkers (alpha ratio and mask score) for all subjects (exploratory and validation) who slept versus those who did not sleep during the EEG recording session. We found no significant differences within the ASD and control groups. Between groups (ASD versus control) for the “sleep” and “did-not-sleep” conditions, we found that the validated results persisted. In addition, we note that maximum power in the alpha band of the 4 posterior electrode deviations was significantly higher in the ASD subjects compared to the control subjects, whether subjects who did not sleep were included (*p* = 0.0015) or excluded (*p* = 0.033) from the analysis. These results do not provide evidence that the presence of sleep during the EEG recording session affected the alpha band findings. We note that these results are consistent with our previous findings that stable functional networks in the EEG persist across different states of consciousness [64].

#### Addressing high frequency myogenic artifact contamination

Electromyogram (EMG) contamination has been shown to impact the EEG at all frequencies [76], particularly in the beta frequency range (i.e., 14 – 30 Hz) and above. To evaluate for possible EMG contamination, we calculated the validated mask score measure for all subjects with data bandpass filtered between 1–10 Hz. We found that the mask score remained significantly different between groups (*p* = 0.0016), indicating that this network biomarker was not driven by myogenic artifact.

#### Addressing low frequency myogenic artifact contamination

An important concern in the analysis of scalp EEG data is the presence of muscle artifact. To assess the impact of muscle artifact, we computed the slope of the logarithmic power versus logarithmic frequency in the four frontal electrode deviations. For normal neuronal population activity, this slope is known to be approximately −2 (e.g., [86]). The effect of broadband muscle artifact is to increase this slope, i.e., to “flatten” the power spectrum. Computing this slope for the exploratory population from 1–15 Hz (e.g., including low frequencies through the alpha band), we find that for each subject and frontal electrode, the slope ranges between [−3, −1.7] in ASD subjects, and [−3.3, −1.5] in control subjects. These slope values are consistent with neuronal activity not dominated by muscle artifact. Moreover, we find no significant differences in the slope between the two groups at any of the four frontal electrodes. These results support the conclusions that muscle artifacts are not dominant in the lower frequency band (1–15 Hz), and that the impact of muscle artifact is similar in the ASD and control groups.

#### Application of the weighted phase-lag index produces similar density results

To investigate how an alternate method of coupling analysis impacts the results, we reanalyzed the combined data using the Weighted Phase-Lag Index (WPLI) [87]. We chose this measure because of its utility for minimizing the effects of volume conduction [87]. We focused the WPLI analysis on the alpha frequency range (8–12 Hz), motivated by the spectral analysis results and the requirement of a relatively narrow frequency interval for a meaningful calculation of phase. Using this alternate measure, we found that the validated mask score remained significantly different (*p* = 0.036) between groups (see Additional file 2: Figure S2).

#### Distribution of ASD severity on validated biomarkers

The distribution of ASD severity (see Methods, Subjects and EEG recordings) with regard to the validated biomarkers was also examined. This preliminary analysis - for a limited number of subjects - suggests that the severity of ASD symptoms is correlated with the proposed biomarkers (see Additional file 3: Figure S3). However, future studies utilizing data collected systematically to assess ASD severity are required to further assess the significance of this relationship.

## Discussion

In this manuscript, we described an approach to find and validate electrophysiological biomarkers for the quantitative identification and characterization of autism in children. Using a validation group, we confirmed two hypotheses: the peak alpha ratio is lower in ASD than control subjects, and the connectivity strength of a select group of edges is lower in ASD than control subjects. We also performed a discriminant analysis using the training group to train the classifier, and the validation group to test the classifier, using our two validated hypothesized measures: the peak alpha ratio and the mask density, and found that the classifier was able to successfully identify ASD subjects in the validation data with 83 % accuracy and control subjects with 68 % accuracy. These results suggest that specific and robust electrophysiological biomarkers of ASD exist, and may provide an additional tool for quantitative diagnosis of ASD.

### Spectral features of ASD

Recent EEG studies on the brain rhythms that distinguish ASD from control subjects have produced conflicting reports, in terms of power, brain location, and frequency, including results in the alpha frequency band [4, 16, 43, 79, 80, 88–91]. Changes in brain rhythms associated with ASD remain an active area of research, and understanding the reasons for differences in the reported changes remains a challenge. Analysis of subtle disorders such as ASD using brain imaging is known to be confounded by many artifacts [92]. Different results in the reported literature may be due to differences in task, subject population demographics, choice of EEG reference [54], or methodology. Low statistical power in EEG studies, caused by too few subjects, may also impact the reported results [92, 93]. Moreover, the broad category of ASD may include subject populations with qualitatively different neurophysiology, and therefore result in the study of qualitatively different phenomenon. The consequent difficulties of comparing results across studies highlights the utility of studies with high subject numbers and within study validation of hypotheses.

In this study, using data from both an exploratory dataset group and validation test group obtained during unconstrained states, we found no significant differences in mean power in any frequency band between ASD and control groups. However, we show that the peak alpha ratio, which represents the anterior-posterior alpha gradient, is significantly lower in the ASD group than the control group, in both the training and validation populations.

Higher frontal power and lower posterior power has also been observed in relation to higher behavioral inhibition and lower sociability [58]. While this measure has not been directly related to ASD, abnormal social interactions is one of the main behavioral traits used to diagnose autism [1]. In addition, a higher alpha gradient is observed in the behavioral state of quiet wakefulness and with brain maturation [94]. Our findings may reflect immaturity in cortical rhythms or the decreased ability of ASD patients to generate this behavioral state. The reproducibility and significance of the changes in alpha band activity reported here suggest this measure may serve as a reliable biomarker of ASD. Future studies including detailed behavioral assessments are needed to determine if alterations in this cortical measure correlate with specific behavioral symptoms.

### Network features of ASD

Much recent work has focused on the inference and analysis of network structure in ASD [95, 96]. Typically, this work has analyzed the anatomical connections (i.e., the “structural network”) between brain regions. The analysis of “functional networks” inferred from scalp EEG data of ASD subjects is relatively sparse ([80, 91, 97, 98] for example). Functional networks are presumed to reveal the transient patterns in communication between brain regions [22]. Because ASD has been postulated to be a disorder of communication between various brain regions, analysis of functional networks is a natural choice. The most frequently reported finding has been lower widespread network connectivity, and occasionally higher local connectivity in specific locations (EEG: [40, 77, 78, 89], fMRI: [16, 18, 88], MEG: [79, 81]). However, contradictory findings have also been reported (EEG: [36, 44, 45, 47], fMRI: [50, 91], MEG: [79], Other/Multiple modalities: [80, 99, 100]). Specifically, recent work has reported *higher* long range connectivity in ASD subjects (fMRI: [43, 46], Other/Multiple modalities: [80]). Connecting EEG network findings to behavior and pathology remains an active research challenge. To that end, lower long-range connectivity in ASD has been related to clinical symptoms such as reduced capacity to integrate brain areas needed for task performance and socialization, while higher local connectivity has been related to an increased focus on specific tasks that is seen in the obsession with repetitive behaviors (fMRI: [17, 101] MEG: [81]). However, further research is required to establish definitive relationships between alterations in EEG functional networks and specific behavioral profiles.

In the exploratory phase of our study, we found that overall connectivity, as measured by the density of functional networks inferred using the cross correlation was significantly lower in the ASD group than the control group, consistent with reported results in the literature. However, this finding was not reproduced in a subsequent validation study, highlighting the uncertainty of the initial results. Although we carefully selected patients and EEG segments for analysis, potential explanations for this lack of validation include the considerable measurement noise inherent in EEG, the diversity of characteristics inherent in ASD, and the choice of coupling analysis parameters. A measure of density that targets specific edges revealed significantly lower connectivity in the ASD subjects that was confirmed in the validation study. We therefore hypothesize that the proposed spatially focused analysis is a more sensitive measure, potentially omitting non-relevant brain activity that may obfuscate the differences between the subject groups.

### An EEG classifier for ASD

A primary goal of this work was to use the scalp EEG to propose a biomarker for ASD. Using a common quadratic discriminant analysis, trained on the training group of subjects and tested on the validation group, but excluding Asperger’s subjects we classified 83 % of ASD subjects and 68 % of control subjects correctly. There have been few previous attempts to classify ASD subjects based on EEG data [7, 40, 97, 102]. Combined with existing diagnosis procedures, a biomarker deduced from scalp EEG would provide an additional cost-effective and relatively straightforward procedure to improve ASD diagnosis. Ultimately, a deep understanding of ASD will require insight into the biological and neurological mechanisms of the disease. However, new biomarkers may immediately help clinicians both diagnosis ASD and assess the severity. Although we did not consider severity of ASD symptoms in the analysis, we did find a broad range of deduced measure values within the ASD population. In the peak alpha ratio and selected edge subset weight there was considerable variation within groups. Moreover, we note that the ASD subjects diagnosed with Asperger’s syndrome tended to have peak alpha ratios and mask density values closer to the mean control group values than the mean ASD group values (Fig. 5). This indicates the possibility that the proposed biomarkers, and perhaps others, could be parametrically related to the severity of ASD symptoms. To further test the relationship between metrics inferred from electrophysiological data and disease severity remains a topic of continuing study. A complete understanding of this relationship would benefit from additional clinical diagnosis relating quantitative measurements to severity of behavior and behavioral test scores, behavioral analysis that includes the control population, and more specific behavioral tasks during recording.

## Conclusions

The field of quantitative analysis of brain activity through spectral and network analysis is a promising one. Recently, more research has focused on the application of these tools to electrophysiological signals for characterization of ASD [80]. In this work, we separated two large subject pools for the exploration of hypotheses and the subsequent validation of these hypotheses with a completely naïve testing population. This study has the benefit of a large subject pool, and represents one of very few EEG studies conducted on children with ASD, when the condition still has the best possible prognosis for amelioration [6]. We also used principled methods to mitigate the impact of common challenges in EEG research, including the effects of volume conduction and reference effects. However this study was limited by a lack of recorded severity scale of ASD or socio-behavioral analysis for all subjects, which might relate the level of the proposed biomarkers to the severity of the symptoms. In addition, the clinical data consisted of spontaneous behavior, which means that no task was performed, and subject movement was not stringently controlled. This lack of behavioral constraint invariably adds noise to the data, but also has the potential benefit of making the results more widely applicable in clinical use. We evaluated clinical EEGs obtained under standardized recording settings for all subjects. However, it is possible that differences in vigilance between ASD and control groups could affect the findings. Finally, we note that subjects diagnosed with Asperger’s disease were not removed from the analysis, which introduces an additional variability into the ASD subject population.

In general, brain imaging research in ASD suffers from many significant sources of potential discrepancy and ambiguity, so it becomes essential for studies to be conducted with high numbers of subjects and within study validation. In particular, because different convergent causes (genetically or neuro-anatomically) may result in similar behavioral outcomes, establishing a specific relationship between imaging or biological metrics with behavioral diagnostic tests that measure severity of ASD symptoms, from the most severely affected and through the normal population [103, 104], may provide new insights. Efforts to standardize the development of diagnostic testing in psychiatry are already underway, as performed by Arfken et al. [105, 106], who use EEG findings from the literature to present a systematic approach for standardizing biomarkers into clinically useful diagnostic screens; a similar approach could potentially be applied here. This study represents a step in the direction of finding neuroimaging metrics that clinicians may use to diagnose ASD, and potentially measure severity and decide on proper treatment approach.

We have demonstrated two biomarkers, an alpha frequency power measure and a subset of edges, that significantly differentiate between a population of ASD and control subjects, and which were validated within study. Although these population indicators do not provide a definitive diagnosis of ASD, they do provide complementary quantitative tools for clinicians to supplement existing diagnosis criteria. Moreover, these results further support the utility of quantitative EEG analysis in the diagnosis of ASD.

## Additional files

Additional file 1: Figure S1. Cross correlation network density using Transverse Bipolar and Hjorth-Laplacian reference montages. Cross correlation network density was examined using transverse bipolar (top) and Hjorth-Laplacian (bottom) reference montages as well as longitudinal bipolar for wake and sleep data (wake data shown). The results found were qualitatively similar to those found using the longitudinal bipolar (double banana) montage. Additional file 2: Figure S2. Application of the weighted phase-lag index produces similar density results. WPLI analysis on the alpha frequency range (8-12 Hz). (Left) Full network density of ASD (blue) and control (red) groups is not significantly different (*p* > 0.1). (Right) Mask density reveals a significant difference (*p* = 0.036) between the ASD group (blue) and the control group (red). Additional file 3: Figure S3. Analysis of biomarkers as a function of ASD severity. A scatter plot of ASD subjects coded by severity (black = severe ASD, blue = moderate ASD, green = mild ASD) and control subjects (red) illustrates a possible correlation between severity and the biomarkers of mask score (vertical axis) and alpha ratio (horizontal axis) found in this study.

## Abbreviations

ASD: Autism spectrum disorders
EEG: Scalp electroencephalogram
FDR: False detection rate
fMRI: functional Magnetic resonance imaging
NREM: Nonrapid eye movement
QDA: Quadratic discriminant analysis
