# Erratum: Robust disruptions in electroencephalogram cortical oscillations and large-scale functional networks in autism

**DOI:** 10.1186/s12883-015-0391-4

**Published:** 2015-08-19

**Authors:** Sean Matlis, Katica Boric, Catherine J. Chu, Mark A. Kramer

**Affiliations:** Graduate Program in Neuroscience, Boston University, 677 Beacon st., Boston, 02215 MA USA; Department of Neurology, Massachusetts General Hospital, 175 Cambridge St., Ste 340, Boston, 02114 MA USA; Harvard Medical School, Boston, 02115 MA USA; Department of Mathematics and Statistics, Boston University, 111 Cummington Mall, Boston, 02215 MA USA

## Erratum

The original version of this article unfortunately contained a mistake. The presentation of Figs. 1 and 2 and their associated figure legends was incorrect in the HTML and PDF versions of this article. The corrected versions are given below. The original article was corrected to reflect this change [1].

**Fig. 1:**
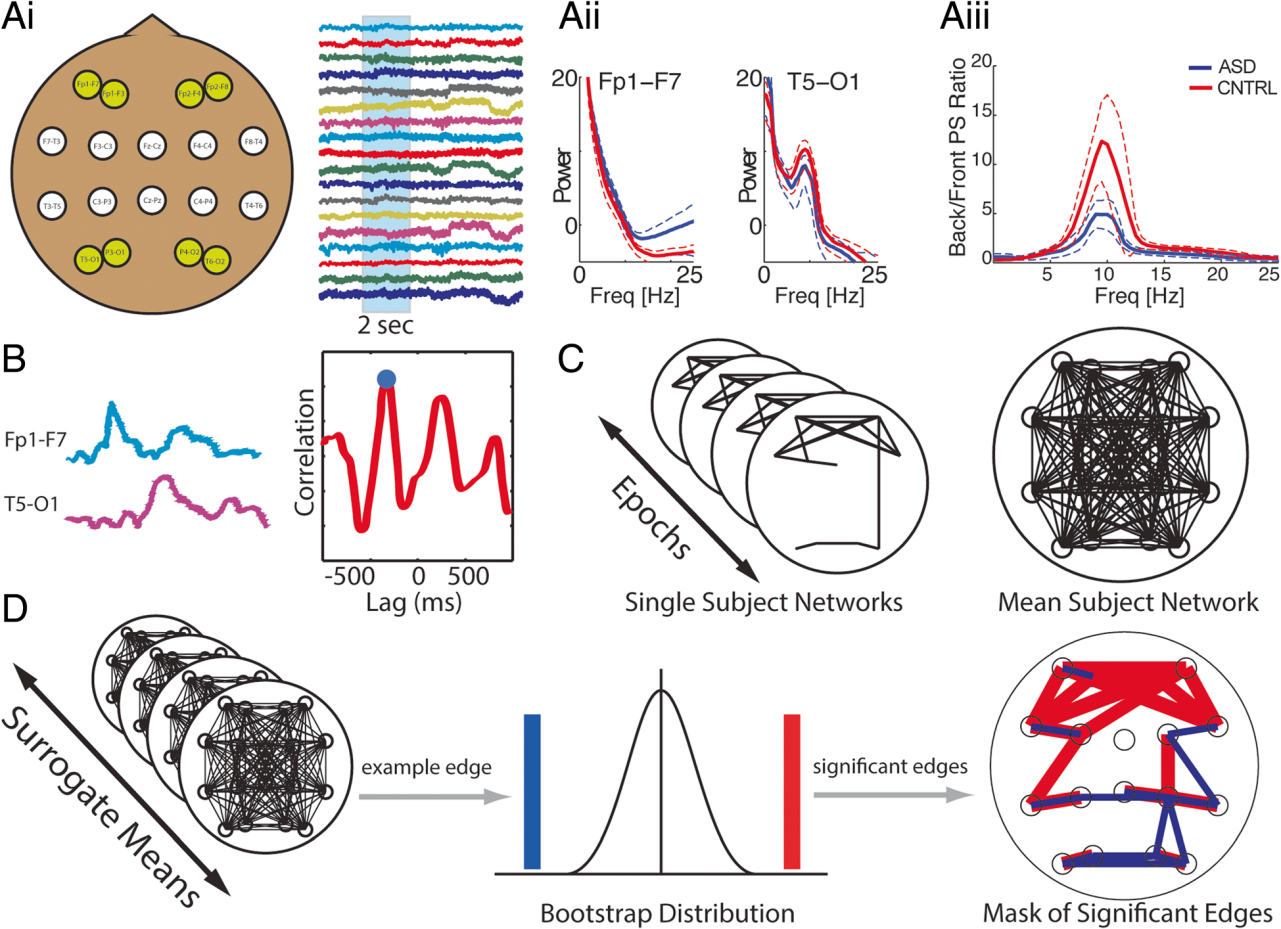
Significant electrode coupling is represented with an edge

**Fig. 2:**
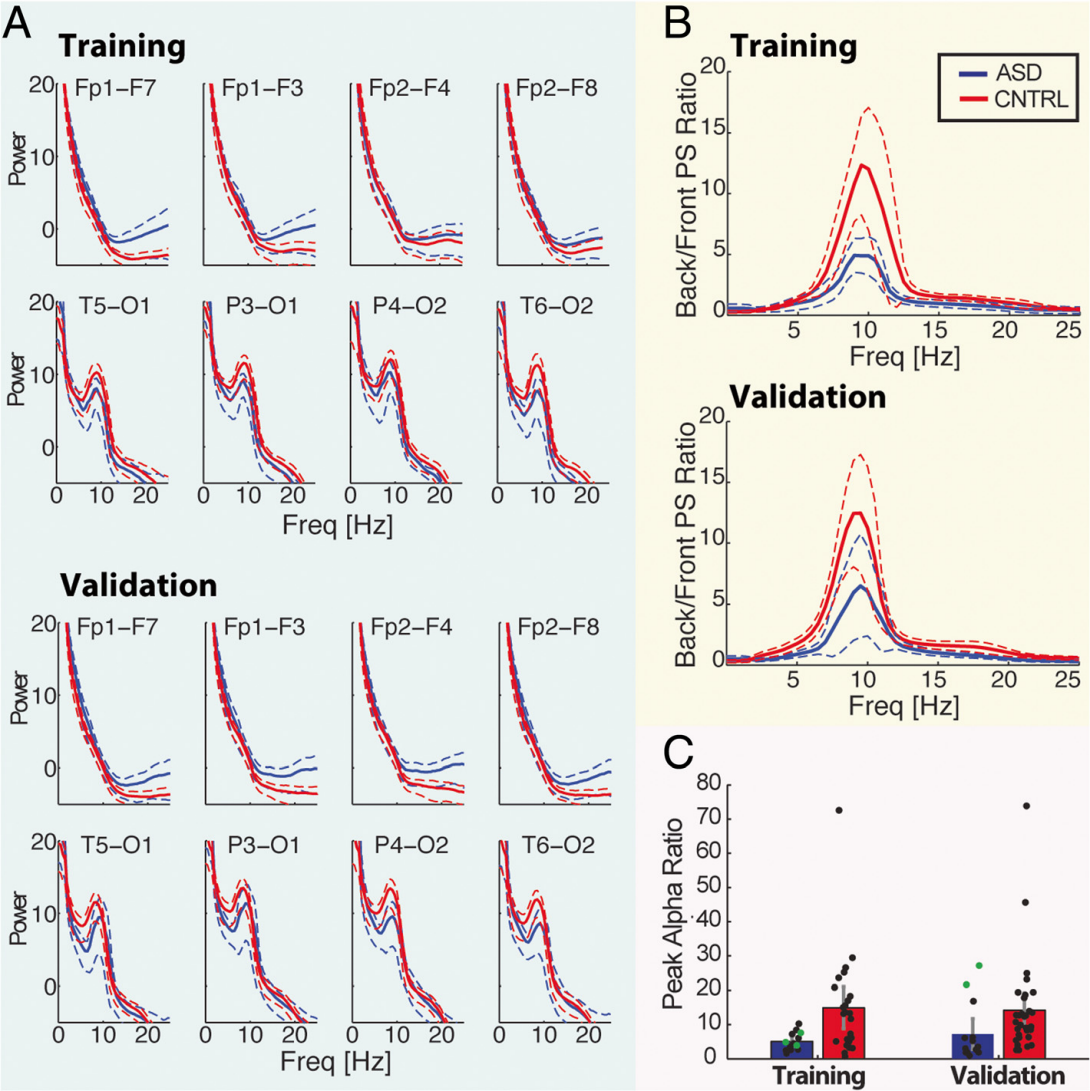
Dashed lines represent two standard errors of mean
