# Supplementary material for: Robust disruptions in electroencephalogram cortical oscillations and large-scale functional networks in autism
Source: BMC Neurol. 2015 Jun 27;15:97. doi: 10.1186/s12883-015-0355-8 (PMC4482270; doi:10.1186/s12883-015-0355-8)
Supplement: Additional file 1: Figure S1. — Cross correlation network density using Transverse Bipolar and Hjorth-Laplacian reference montages. Cross correlation network density was examined using transverse bipolar (top) and Hjorth-Laplacian (bottom) reference montages as well as longitudinal bipolar for wake and sleep data (wake data shown). The results found were qualitatively similar to those found using the longitudinal bipolar (double banana) montage. [file 12883_2015_355_MOESM1_ESM.pdf]

SUPPLEMENTARY MATERIAL:

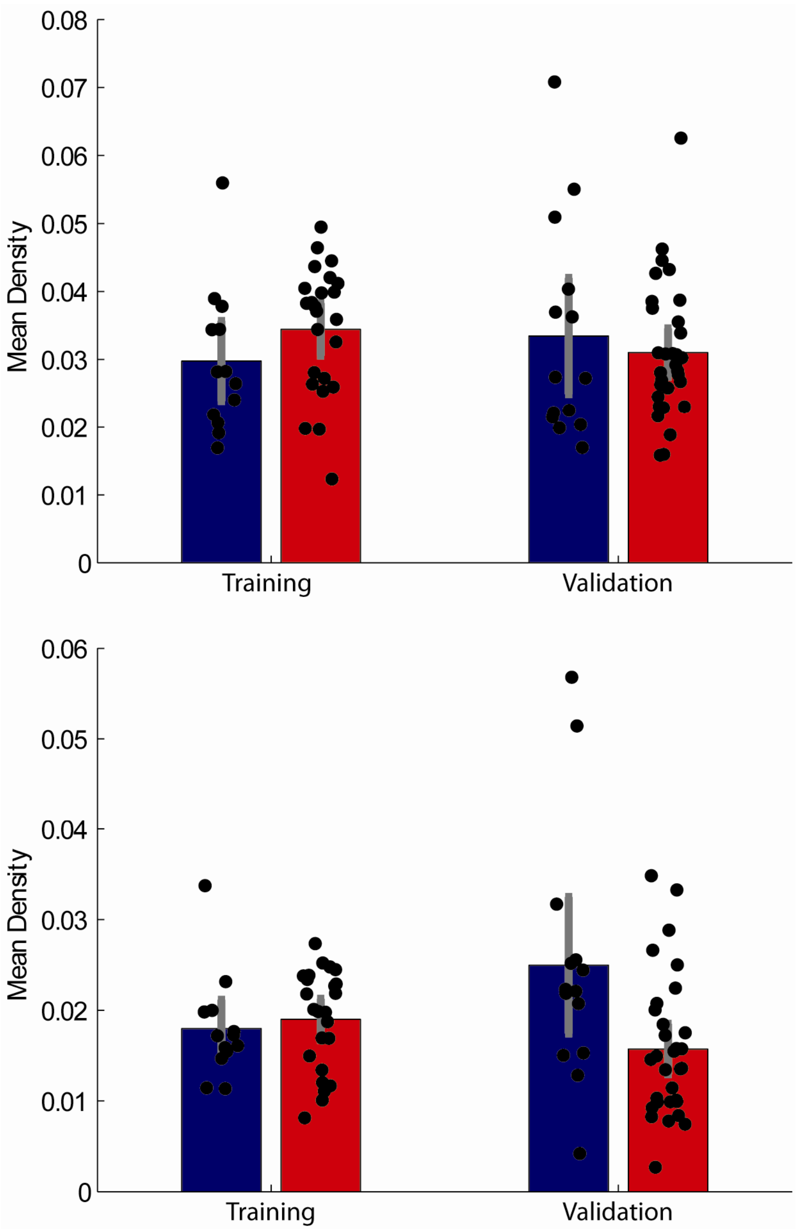

Supplementary Fig 1. Cross correlation network density using Transverse Bipolar and Hjorth-Laplacian reference montages. *Cross correlation network density was examined using transverse bipolar (top) and Hjorth-Laplacian (bottom) reference montages as well as longitudinal bipolar for wake and sleep data (wake data shown). The results found were qualitatively similar those found using the longitudinal bipolar (double banana) montage.*
