# Supplementary material for: Robust disruptions in electroencephalogram cortical oscillations and large-scale functional networks in autism
Source: BMC Neurol. 2015 Jun 27;15:97. doi: 10.1186/s12883-015-0355-8 (PMC4482270; doi:10.1186/s12883-015-0355-8)
Supplement: Additional file 2: Figure S2. — Application of the weighted phase-lag index produces similar density results. WPLI analysis on the alpha frequency range (8-12 Hz). (Left) Full network density of ASD (blue) and control (red) groups is not significantly different (p > 0.1). (Right) Mask density reveals a significant difference (p = 0.036) between the ASD group (blue) and the control group (red). [file 12883_2015_355_MOESM2_ESM.pdf]

WPLI Analysis

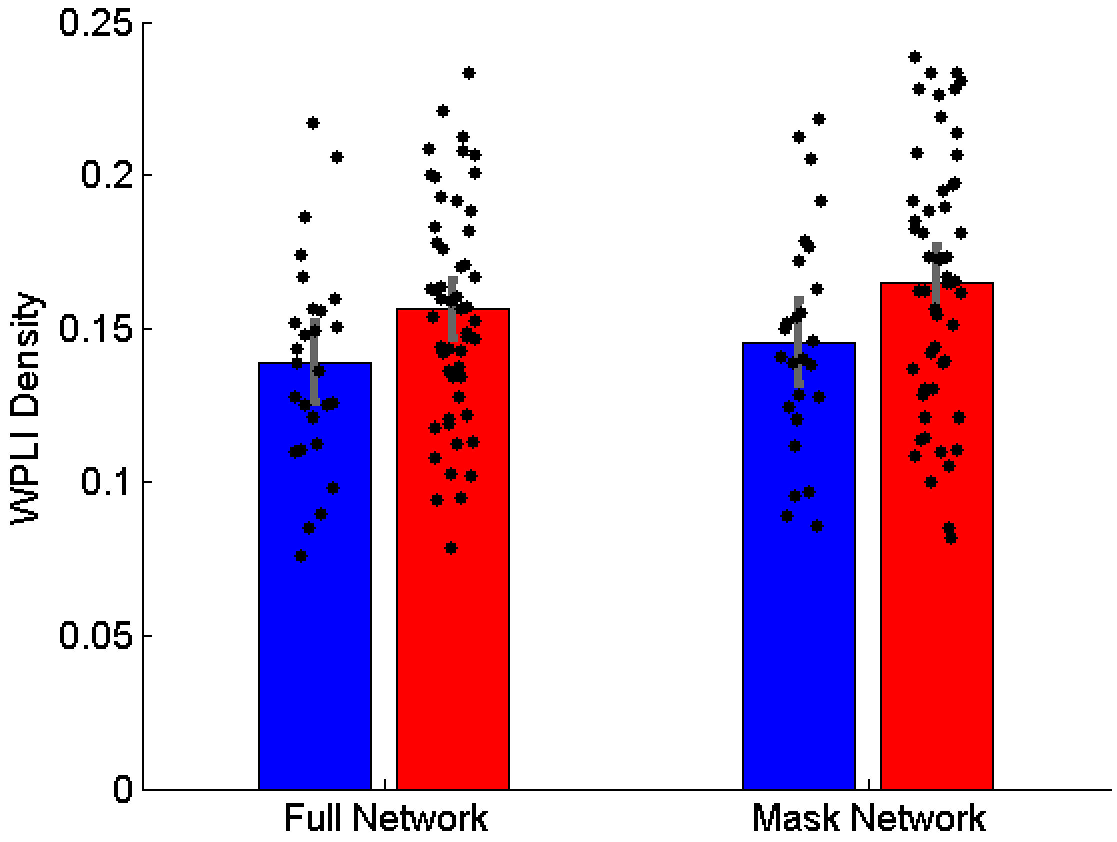

Supplementary Fig 2. Application of the weighted phase-lag index produces similar density results. *WPLI analysis on the alpha frequency range (8-12 Hz). (Left) Full network density of ASD (blue) and control (red) groups is not significantly different ($p > 0.1$). (Right) Mask density reveals a significant difference ($p=0.036$) between the ASD group (blue) and the control group (red).*
