# Supplementary material for: Robust disruptions in electroencephalogram cortical oscillations and large-scale functional networks in autism
Source: BMC Neurol. 2015 Jun 27;15:97. doi: 10.1186/s12883-015-0355-8 (PMC4482270; doi:10.1186/s12883-015-0355-8)
Supplement: Additional file 3: Figure S3. — Analysis of biomarkers as a function of ASD severity. A scatter plot of ASD subjects coded by severity (black = severe ASD, blue = moderate ASD, green = mild ASD) and control subjects (red) illustrates a possible correlation between severity and the biomarkers of mask score (vertical axis) and alpha ratio (horizontal axis) found in this study. [file 12883_2015_355_MOESM3_ESM.pdf]

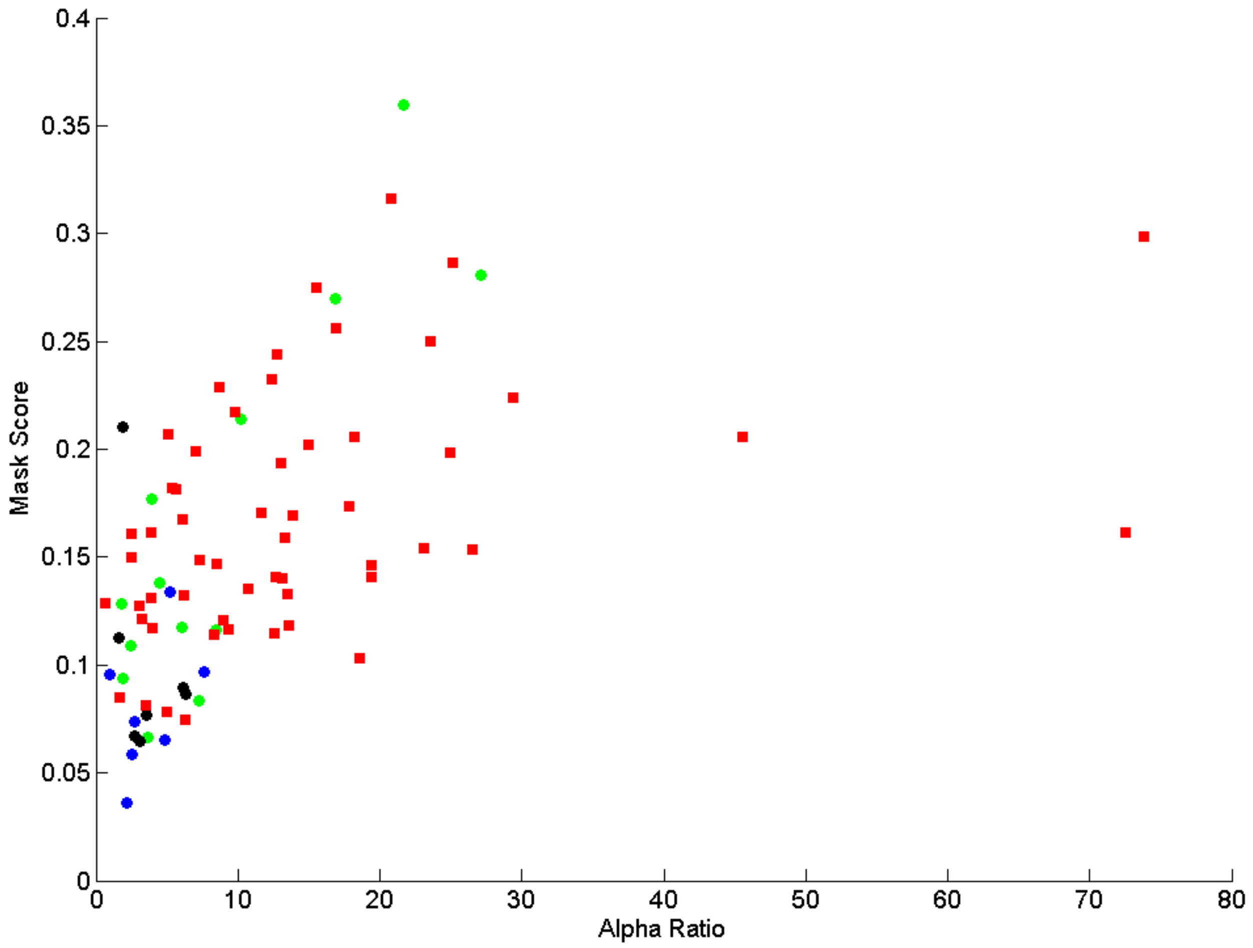

Supplementary Fig 3. Analysis of biomarkers as a function of ASD severity. *A scatter plot of ASD subjects coded by severity (black = severe ASD, blue = moderate ASD, green = mild ASD) and control subjects (red) illustrates a possible correlation between severity and the biomarkers of mask score (vertical axis) and alpha ratio (horizontal axis) found in this study.*
